# Therapeutic approaches to pediatric COVID-19: an online survey of pediatric rheumatologists

**DOI:** 10.1007/s00296-021-04824-4

**Published:** 2021-03-08

**Authors:** Ales Janda, Catharina Schuetz, Scott Canna, Mark Gorelik, Maximilian Heeg, Kirsten Minden, Claas Hinze, Ansgar Schulz, Klaus-Michael Debatin, Christian M. Hedrich, Fabian Speth

**Affiliations:** 1 Department of Pediatrics and Adolescent Medicine, Ulm University Medical Center, Eythstrasse 24, 89075 Ulm, Germany; 2 Department of Pediatrics, Medizinische Fakultät Carl Gustav Carus, Technische Universität Dresden, Dresden, Germany; 3 University of Pittsburgh, Pittsburgh, PA USA; 4 Department of Pediatrics, Division of Allergy, Immunology, and Rheumatology, Columbia University Irving Medical Center, New York Presbyterian Morgan Stanley Childrens Hospital of New York, New York, NY USA; 5 Institute for Immunodeficiency, Center for Chronic Immunodeficiency (CCI), Medical Center - University of Freiburg, Faculty of Medicine, University of Freiburg, Freiburg, Germany; 6 Charité University Medicine Berlin, German Rheumatism Research Center Berlin, Berlin, Germany; 7 University Hospital Munster, Munster, Germany; 8 Department of Women’s & Children’s Health, Institute of Life Course and Medical Sciences, University of Liverpool & Department of Paediatric Rheumatology, Alder Hey Childrens NHS Foundation Trust Hospital, Liverpool, Great Britain; 9 Department of Pediatric Rheumatology, Pediatric Bone Marrow Transplantation and Immunology Unit, Center for Obstetrics and Pediatrics, University Medical Center Hamburg-Eppendorf, Hamburg, Germany

**Keywords:** COVID-19, SARS-CoV-2, Children, Pediatric rheumatology, Autoimmune disease, Inflammation, Treatment, Opinion poll

## Abstract

Data on therapy of COVID-19 in immunocompetent and immunosuppressed children are scarce. We aimed to explore management strategies of pediatric rheumatologists. All subscribers to international Pediatric Rheumatology Bulletin Board were invited to take part in an online survey on therapeutic approaches to COVID-19 in healthy children and children with autoimmune/inflammatory diseases (AID). Off-label therapies would be considered by 90.3% of the 93 participating respondents. In stable patients with COVID-19 on oxygen supply (stage I), use of remdesivir (48.3%), azithromycin (26.6%), oral corticosteroids (25.4%) and/or hydroxychloroquine (21.9%) would be recommended. In case of early signs of “cytokine storm” (stage II) or in critically ill patients (stage III) (a) anakinra (79.5% stage II; 83.6% stage III) or tocilizumab (58.0% and 87.0%, respectively); (b) corticosteroids (oral 67.2% stage II, intravenously 81.7% stage III); (c) intravenous immunoglobulins (both stages 56.5%); or (d) remdesivir (both stages 46.7%) were considered. In AID, > 94.2% of the respondents would not support a preventive adaptation of the immunomodulating therapy. In case of mild COVID-19, more than 50% of the respondents would continue pre-existing treatment with immunoglobulins (100%), hydroxychloroquine (94.2%), anakinra (79.2%) or canakinumab (72.5%), or tocilizumab (69.8%). Long-term corticosteroids would be reduced by 26.9% (< = 2 mg/kg/d) and 50.0% (> 2 mg/kg/day), respectively, with only 5.8% of respondents voting to discontinue the therapy. Conversely, more than 75% of respondents would refrain from administering cyclophosphamide and anti-CD20-antibodies. As evidence on management of pediatric COVID-19 is incomplete, continuous and critical expert opinion and knowledge exchange is helpful.

**Supplementary Information:**

The online version contains supplementary material available at 10.1007/s00296-021-04824-4.

## Introduction

The newly emerged coronavirus SARS-CoV-2 (severe acute respiratory syndrome coronavirus 2) is the infectious pathogen causing COVID-19 (corona virus disease 2019), a pandemic that poses a threat to millions of lives and, therefore, a challenge to healthcare providers of all medical specialities. It became apparent that the shared expertise of rheumatologists and clinical immunologists is critical in the treatment of COVID-19 patients, as a significant proportion of adult and few pediatric patients develop hyperinflammatory disease [1–7].

While the course of COVID-19 in healthy children is usually asymptomatic or mild when compared to adults [8, 9], a hyperinflammation syndrome (pediatric inflammatory multisystem syndrome: PIMS or MIS-C: multisystem inflammatory syndrome in children) timely associated with SARS-CoV-2 has been described in a subset of pediatric patients in Europe and North America [2, 10–13]. Although patients with pediatric autoimmune/inflammatory disease (AID) may be more susceptible to virus infections when compared to age-matched healthy cohorts, and AID patients-receiving immunomodulatory or immunosuppressive therapy are considered to be more susceptible towards viral and bacterial infections [14, 15], there is no reliable evidence to suggest a higher risk of SARS-CoV-2 infection or more severe disease in those patients [4, 16–21]. Whether immune deficiency and/or immunomodulating treatment may even prevent severe complications such as PIMS-TS/MIS-C remains unclear [22]. Thus, in the absence of prospective studies in large cohorts, management of patients-receiving immunosuppression/modulation remains a particular challenge, as data are preliminary and somewhat conflicting as to whether or which type of immunomodulation represents a risk or protective factor for developing hyperinflammatory complications [1, 3, 4, 23, 24]. Published COVID-19-related recommendations providing clinical guidance for the management of children and adolescents [25–31] are largely based on case series, retrospective cohort studies, pathophysiological considerations, or are derived from data on adult populations. As low-quality evidence and expert opinion prevail, continuous inter-collegial discussion facilitate clinical decision making.

Here, we present results of an online opinion poll among international pediatric rheumatologists on: (a) possible general therapy concepts in COVID-19 in children and adolescents and (b) clinical management of pediatric patients with AID-receiving immunomodulation treatment in the context of SARS-CoV-2 pandemic.

## Methods

The survey collecting opinions within the international pediatric rheumatology community was conducted in line with the published recommendations for survey-based research [32].

Pediatric rheumatology professionals (1849 unique email addresses), subscribers to the international online Pediatric Rheumatology Bulletin Board administered at McMaster University in Canada (https://mailman.mcmaster.ca/mailman/listinfo/ped-rhe-list), were invited to participate in the survey via an email distribution list. The survey form was put online using SurveyMonkey ® (https://www.surveymonkey.com) and was open for response from May 28 to June 11, 2020. The participation was voluntary, no incentives were used. One reminder email was distributed. The exact wording of the online survey form is available at Supplementary Figure S1.

**Fig. 1:**
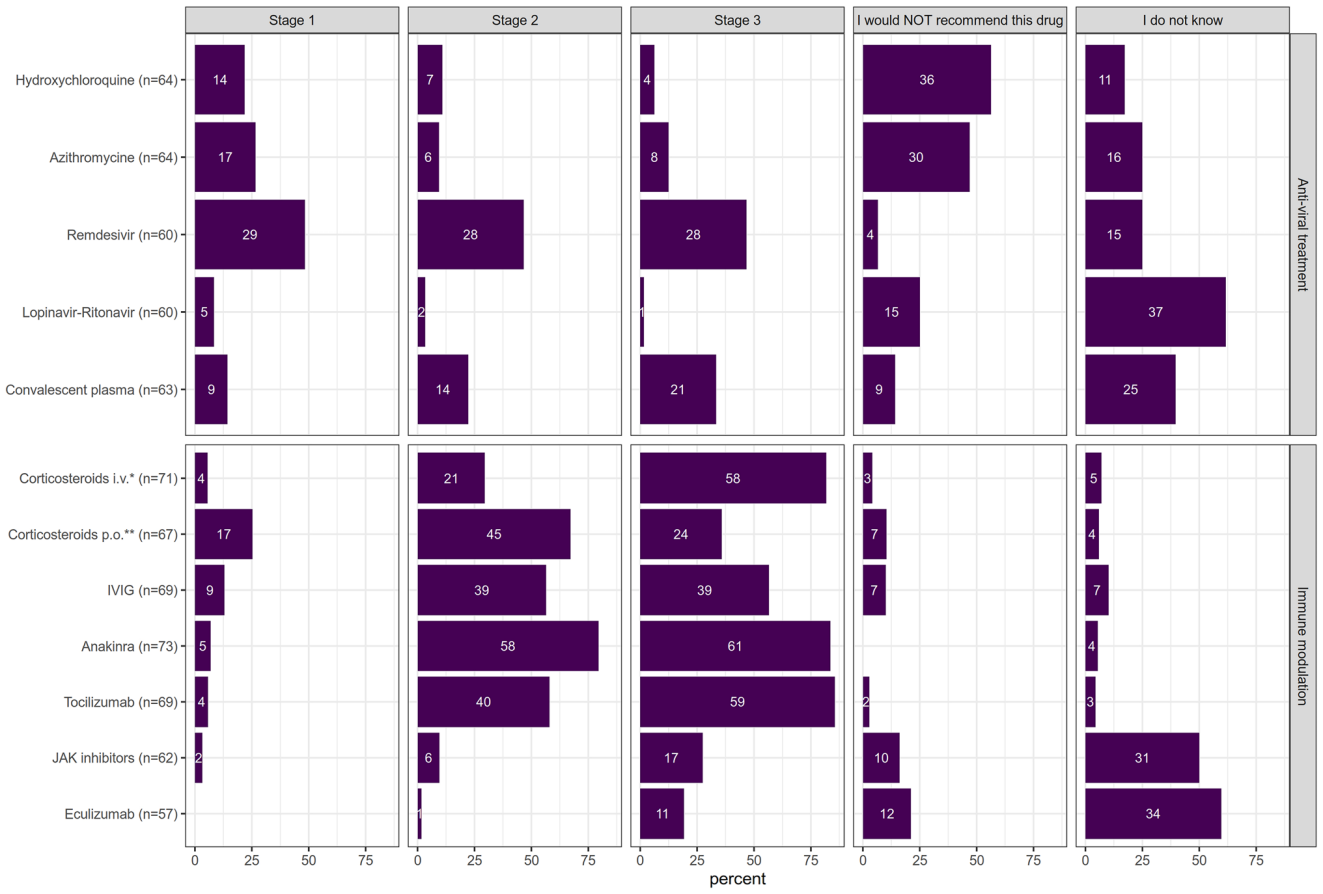
Therapeutics considered for pediatric COVID-19. The number of respondents expressing their opinion on each treatment option is depicted in each line next to the drug name; number of respondents supporting the particular treatment is shown within the bars; their length reflects proportion of positive responses in case of the given treatment. For better comprehension, the treatment options are split into two main categories (anti-viral and immunomodulatory therapy), though the therapeutic effects might be overlapping. *prednisolone ≤ 2 mg/kg/d, **high-dose prednisolone (10–30 mg/kg/d)

Data on respondents’ institutions, their experience in pediatric rheumatology (in years) and their practical experience with the care of COVID-19 patients were collected. Respondents could express their readiness to consider use of (off-label) therapies in COVID-19-affected cases. A list of suggested drugs (see Fig. 1) was provided in alphabetical order. Potential treatment choices were available for in-patients stratified by COVID-19 disease severity:

(i) Stage I: pneumonia with oxygen demand; (ii) Stage II: additional respiratory deterioration and/or imminent cytokine storm; (iii) Stage III: a critically ill patient with acute respiratory distress syndrome or multiorgan failure.

Respondents could express their disapproval with the use of a particular drug at any stage of the disease (“I would not recommend this drug”). In case of uncertainty, a statement “I do not know” could be selected. In case of treating a patient with an increased risk of severe viral disease (e.g. a patient with relevant immunodeficiency or severe cardiopulmonary, nephrological or neurodegenerative underlying disease), respondents were asked whether they would choose the following therapies earlier in the course of the disease: (a) antiviral drugs; (b) corticosteroids; (c) immunomodulatory drugs; (d) cytokine blockade; (e) immunoglobulins; or (f) convalescent plasma.

Treatment options for PIMS/MIS-C were not specifically addressed in this survey.

**Fig. 2:**
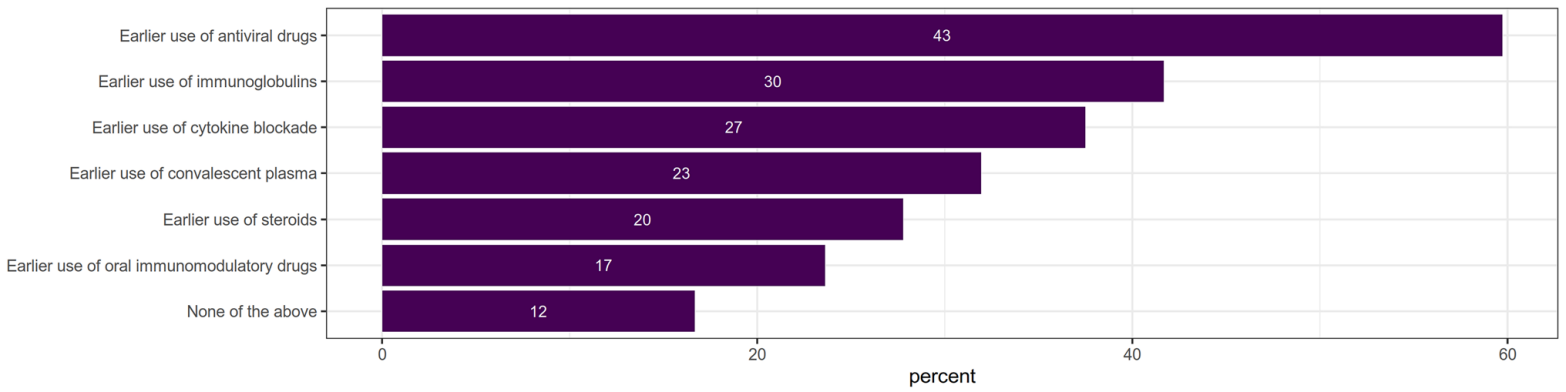
Opinion on the possible earlier use of particular treatment modalities in course of COVID-19 in patients with increased risk for severe course of viral infections (*n* = 72 respondents). Number of respondents supporting the given approach is shown at each bar; its length reflects proportion of positive responses

In the second part of the survey, the approach to anti-inflammatory, immunomodulatory/immunosuppressive therapy in patients with AID in the context of COVID-19 was addressed. First, respondents could report on their attitude towards preventive adaptation of the ongoing treatment for children with AID in the absence of SARS-CoV-2 exposure or infection. Second, respondents’ treatment strategies in patients with mild symptoms of proven COVID-19 were addressed (list of the drugs in Fig. 2). Possible response options included “no change”, “reducing” or “stop” the long-term medication.

Close-ended answer choices were applied in all questions. The online survey form was subjected to internal validation. Data anonymity were warranted.

### Statistical analysis

Only complete datasets (including respondents’ characteristics, responses from part one and/or two) were subjects to analysis. Statistical analysis was performed using R (v 4.0.0) and the tidyverse packages dplyr (v 1.0.2). Graphics were generated using the R package ggplot2 (v 3.3.2). Double-sided Fisher's exact test was applied to compare frequencies of particular responses between different participant groups.

### Ethics statement

The study was exempt from ethical review while no patients or study subjects were involved. Only anonymous demographic and expert opinion data were collected. The respondents were made aware in the text of the invitation email that the results of the survey will be eventually published.

## Results

### Respondents’ characteristics

Ninety-three members of the mailing list from 23 countries responded (5% of all contacted individual email accounts) (Table 1). The majority of respondents worked in the U.S.A (50.5%; Supplementary Table 1, Supplementary Figure S2), most of them at university hospitals (88.2%). Over half of the respondents were senior healthcare professionals working in the field of pediatric rheumatology for over 10 years (52.7%). About two-thirds indicated personal experience with the care of patients with COVID-19.

**Table 1:** Respondents’ characteristics

Type of medical setting (*n* = 93)	
University hospital	82 (88.2%)
Other hospital	10 (10.8%)
Out-patient setting	1 (1.1%)
Geographical location (*n = *90)	
Americas	60 (64.5%)
Europe	24 (25.8%)
Asia	4 (4.3%)
Africa	1 (1.1%)
Australia	1 (1.1%)
Experience in the field of pediatric rheumatology (*n* = 92)	
< 5 years	12 (13.0%)
5–10 years	31 (33.3%)
> 10 years	49 (52.7%)
Experience with treatment of patients with COVID-19 (*n* = 92)	
Yes	58 (63.0%)
1–5 patients	33 (35.5%)
> 5 patients	25 (26.9%)
Pediatric patients	47 (50.5%)
Adult patients	2 (2.2%)
All age categories	8 (8.7%)
No	34 (36.6%)

### Treatment strategies in pediatric patients with COVID-19

Eighty-four respondents (90.3%) would consider off-label treatment in COVID-19 (Fig. 1).

The first three most commonly suggested therapeutics for patients in stage I of COVID-19 were the ribonucleotide analogue remdesivir (48.3%), the macrolide antibiotic azithromycin (26.6%) and the antimalarial agent hydroxychloroquine (HCQ; 21.9%). While HCQ and azithromycin lost support in the stage II (10.9% for HCQ and 9.4% for azithromycin) as well as stage III (6.3% and 12.5%), the use of remdesivir was considered beneficial by a similar proportion of respondents (46.7% for both, stage II and III). Only relatively few respondents (6.7%) would not recommend remdesivir at all. Indecisiveness with respect to the above-mentioned drugs was comparable (17.2% in case of HCQ, 25% azithromycin, and 25% for remdesivir). Notably, a majority of respondents would use neither HCQ (56.3%) nor azithromycin (48.4%) at all. Use of lopinavir–ritonavir would be considered by only few respondents (8.3%; 3.3% and 1.7% for the three disease stages, respectively); the majority were undecided (61.7%) or argued against its use (26.7%). Administration of convalescent plasma gained importance for the respondents throughout the disease progression (14.3%; 22.2% and 35.0%). However, a relatively high proportion of respondents were undecided (40%); only a small minority (14.3%) expressed concern and would not administer convalescent plasma at all.

A significant proportion of respondents would consider *immunomodulatory therapy* in the management of pediatric COVID-19. Oral corticosteroid therapy (prednisolone ≤ 2 mg/kg/d) was the most frequently chosen option (25.4%) in stage I. Its importance to the respondents increased with disease progression (stage II, 67.2%). However, in case of severe disease with cytokine storm and multiorgan failure (stage III) high-dose intravenous corticosteroid therapy (prednisolone 10–30 mg/kg/d) would be preferred (81.7% for the intravenous form versus 35.8% for the oral variant). More than half of the respondents considered administering intravenous immunoglobulins (IVIG) for more advanced disease (56.5% for both stages II and III). A majority of respondents opted for cytokine blocking strategies in imminent or already active hyperinflammation as most important therapeutical approach. Blockade of IL-6 (tocilizumab, 85.5%) or IL-1 (anakinra 83.6%) in stage III was supported by most respondents. Interestingly, in milder disease (stage II), IL-1 blockade was considered more frequently (79.5%) as compared to IL-6 blockade (58.8%). None of the respondents argued against IL-1-blockade; similarly, opposition to IL-6 blockade with tocilizumab was low (3.0%). Janus kinase (JAK) or complement (eculizumab) inhibition attracted the least attention as therapeutic strategy across disease stages, with highest support in stage III (27.4% for JAK-inhibitors and 19.3% for eculizumab). With the exception of JAK and complement inhibition, most respondents expressed confidence in the efficacy of immunomodulatory therapy while the uncertainty fluctuated between 4.3 and 10.1%. For the use of JAK (50%) and complement inhibition (59.6%), however, a majority of respondents were undecided.

Next, we asked whether treatment decisions and choice of particular drugs would depend on the respondents’ experience and/or characteristics. The only significant difference was found for the use of HCQ (*p* = 0.025) and azithromycin (*p* = 0.026). More senior colleagues (> 10 years of experience) would use HCQ and azithromycin more readily (in stage I) and were generally less critical of these options.

### Treatment approaches in potentially immunocompromised patients

When comparing responses concerning treatment of patients with increased risk for viral infections with those of otherwise healthy COVID-19 patients (Fig. 2), our respondents would promote an earlier use of antiviral drugs (59.7% of the respondents), followed by use of immunoglobulins (41.7%), cytokine blockade (37.5%), convalescent plasma (31.9%), corticosteroids (27.8%) and oral immunomodulatory drugs (23.6%) in the course of the disease. Twelve respondents would choose neither of those options.

The choice differences between the participants’ groups were not statistically significant.

### Approach to pre-existing anti-inflammatory, immunomodulatory and immunosuppressive therapy in patients with AID in the context of COVID-19 pandemic

Fifty-eight respondents (62.4%) submitted their responses for this section of the survey. For patients without SARS-CoV-2 infection or signs of COVID-19, 94.2% of those respondents would continue established anti-inflammatory/immunomodulating therapy. Three respondents would consider reduction or discontinuation of an existing long-term therapy in patients with clinically inactive rheumatological disease.

**Fig. 3:**
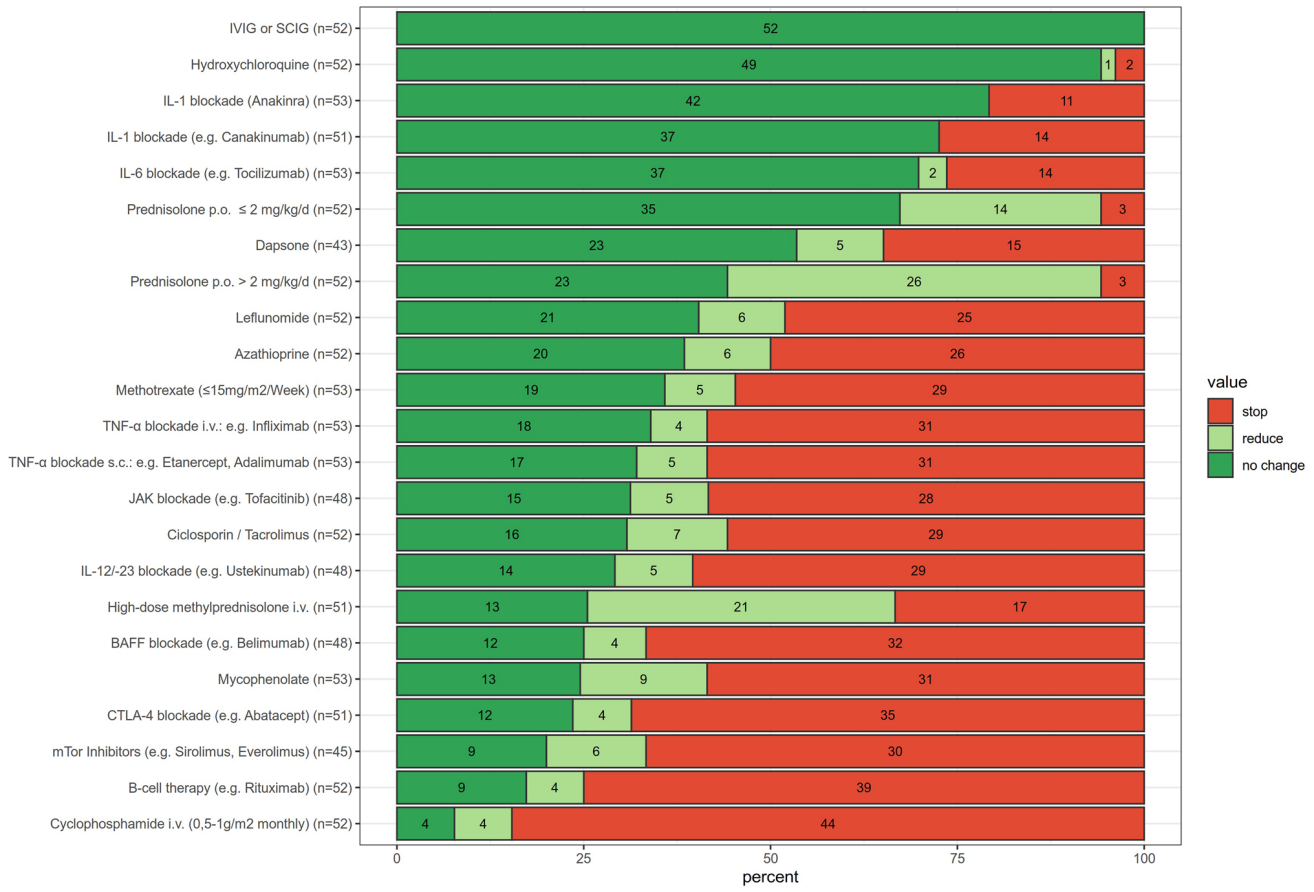
Opinion on how to approach established anti-inflammatory/immunomodulatory treatment in patients with autoimmune/inflammatory disease (AID) and confirmed mild COVID-19 disease. The number of respondents expressing their opinion on each medication is depicted next to the graph; the number of respondents supporting a particular approach is shown for each bar; its length reflects the proportion of positive responses. The treatment options are arranged in descending order with respect to how strongly physicians felt about continuing treatment despite mild COVID-19

In AID patients with proven “mild” (stage I) COVID-19 (Fig. 3) the following therapies would be continued: immunoglobulin therapy (100%), HCQ (94.2%), IL-1 blockade with canakinumab and anakinra (72.5% and 79.2%, respectively) and IL-6 blockade (with support of 69.8% respondents). The majority of the respondents would discontinue or modify (postpone or reduce a dose) therapies with mTOR-inhibitors (80%), CTLA-4-blockade (76.5%), mycophenolate mofetil (75.5%), BAFF-blockade (75%), IL-12/23-blockade (70.8%), calcineurin inhibitors (69.2%), JAK inhibition (68.7%), TNF-α blockade (67.9%), methotrexate (64.2%), azathioprine (61.5%) and leflunomide (59.6%). Treatment with dapsone would be reduced by 46.5% of the respondents. Over 75% of respondents would refrain from initiating therapy with cyclophosphamide (84.6%) or anti-CD20-antibodies (74.9%).

Discontinuation of long-term oral corticosteroid therapy (≤ 2 as well as > 2 mg/kg/d) would only be considered by 5.8% of respondents. However, a majority of respondents would consider dose reduction (26.9% in ≤ 2 mg/kg/d; 50% in > 2 mg/kg/d). As many as 27.5% would discontinue/refrain from intravenous high-dose “pulse” therapy with methylprednisolone, 41.2% would reduce the dose.

The only statistically significant difference between the groups of respondents was found in case of IL-6 blockade with tocilizumab. The majority of senior colleagues (> 10 years of experience in pediatric rheumatology) would recommend maintenance of the IL-6 blockade (83.9%) in comparison to the less experienced colleagues who would endorse this approach in only 50% (*p* = 0.014).

## Discussion

An opinion data of almost one hundred international pediatric rheumatologists at the time of peaking COVID-19 pandemic are presented. Due to the rapid evidence evolvement in these unique times may this survey impose slightly outdated. However, the growing body of evidence regarding treatment options in patients with COVID-19 or management strategies of individuals with AID applies still mainly to the adult population. Furthermore, the care for children and adolescent with COVID-19 as well as the approach to the pediatric patients with AID in context of the COVID-19 pandemic remains controversial and only scarce data on changes in health care of pediatric patients with rheumatic diseases are available [33, 34].

### Treatment strategies in pediatric patients with COVID-19

In this survey, remdesivir was the most frequently considered virus-directed drug in pediatric patients (almost 50% support throughout all disease stages). Frequent concurrent choice of remdesivir and/or cytokine blockade and/or corticosteroid therapy points towards possible combination strategies. This opinion is very well in line with later published data on positive clinical effects in a subset of patients with COVID-19 [35–37]. Those findings resulted in an emergency use authorization from the US Food and Drugs Administration (FDA), including children over 12 years of age and/or weighing more than 40 kg [38], followed by approval in Europe [39]. Early use during the viral replication stage seems to be most beneficial [40, 41].

Within the group of immunomodulatory drugs corticosteroid use gained the highest respondents’ support, reaching 35.8% and 81.7% use of oral and intravenous corticosteroids, respectively, for patients in stage III. This view corresponds with "real life" COVID-19 treatment data worldwide [42] and is underpinned by the findings from the RECOVERY study on use of dexamethasone [43]. However, a precaution is warranted as the application of dexamethasone in this trial improved outcome only in those on respiratory support [43].

Cytokine blocking strategy (blockade of IL-1 and IL-6) was the most often chosen treatment option for patients with clinically severe COVID-19 reaching support of 83.6% and 85.5% respondents, respectively, for use in stage III. This is in line with data from case series and individual case reports [2, 44, 45], at times in combination with high-dose methylprednisolone [46]. The popularity of anakinra may be based on its great potential to control hyperinflammation, but also its excellent safety profile including use in patients with bacterial sepsis and cytokine storm [47]. In addition, anakinra as well as tocilizumab are approved for use in cytokine release syndrome (macrophage activation syndrome) from 8 and 24 months of age, respectively.

The most controversial findings in the survey concern HCQ and azithromycin. Results of a small clinical trial at the onset of the pandemic showed enhancement of the clearance effect on SARS-CoV-2 by addition of azithromycin to HCQ therapy [48]. This controversial study contributed to optimism and broad uncritical endorsement of HCQ and azithromycin in treating COVID-19. However, growing evidence from controlled clinical trials [49–52] did not show conclusive benefit of the therapy. Moreover, cardiotoxicity, namely rhythm disorders, became an increased concern among adult patients [53, 54]. Currently, HCQ and/or azithromycin are not recommended for treatment of COVID-19. Participants of this survey were rather hesitant about using HCQ and/or azithromycin in COVID-19; however, still around a quarter of them would consider their use in the stage I. The dynamics of opinion change and growing skepticism towards these treatment modalities could be demonstrated when compared to a related, but previous survey that we performed among German-speaking pediatric rheumatologists [55].

Recently, it has been shown that the immunocompromised state of patients with primary immunodeficiency is not a predominant factor for severe course of COVID-19 [22]. With exception of deficiency in type I interferon signaling [22, 56] or presence of autoantibodies against interferons [57]. Secondary immunodeficiency due to chemotherapy in pediatric patients treated for malignant disease is associated with longer viral clearance; however, with only minimally increased risk of severe course of COVID-19 [58]. Thus, the current available data do not justify therapy intensification against SARS-CoV-2 infection in all immunocompromised children considered by some of our respondents and support individualized approach.

### Approach to pre-existing anti-inflammatory, immunomodulatory and immunosuppressive therapy in patients with AID in the context of COVID-19 pandemic

Support for the continuation of pre-existing immunomodulatory and/or immunosuppressive therapy in patients with AID without proven COVID-19 documented in the survey is in line with recommendations of the American College of Rheumatology (ACR) [30] as well as the European societies [31, 59] published after closure of this survey.

In the case of an incipient COVID-19 disease, almost all respondents were in favor of continuing ongoing therapy with HCQ and subcutaneous or intravenous immunoglobulins for the underlying rheumatological disease. In fact, there is no evidence for a disadvantage of these drugs in the context of COVID-19 [60]. The majority of colleagues would also continue a pre-existing oral steroid therapy, however, reduce higher dosages beyond 2 mg/kg/d. The ACR recommends continuation of long-term steroid therapy taking into account the risk of potential adrenal cortex insufficiency (with a pre-treatment period of > 14 days) [30]. The majority of respondents argued against immediate termination of IL-1 or IL-6 inhibition. Discontinuation of a short-acting IL-1-targeted therapy such as anakinra, carries the risk of a rebound phenomenon of the underlying (systemic) rheumatic or inflammatory diseases. Moreover, it may trigger a macrophage activation syndrome, a complication was also observed in some COVID-19 patients [61]. Data supporting continuation of IL-1-blockade during mild COVID-19 has been recently published: a mild course of the infection was shown in three children and one adult treated for autoinflammatory disease with IL-1-targeted therapy [62]. After recovery from the infection two out of the three pediatric patients suffered from an increased autoinflammatory activity. Those flares were presumably due to the underlying disease and not related to the IL-1-targeted therapy [62].

A large number of respondents favors a continuation of dapsone, mycophenolate, leflunomide, methotrexate, azathioprine and calcineurin-inhibitors, possibly administered in reduced dosages. Presumably, antiproliferative drugs would be continued for fear of a severe relapse, e.g. in connective tissue diseases and vasculitides. In COVID-19 patients-receiving ongoing immunomodulating/suppressive drugs, close monitoring of blood count changes, liver and kidney toxicity and, parameters such as IgG, IgM and CD4 T-cell numbers are justified [63].

The majority of respondents would pause TNF-alpha inhibitors and IL-12/23-inhibitors in COVID-19 patients. As in other virus infections, this is a standard approach for patients with inactive juvenile (psoriatic) arthritis due to the fast effect at restart of TNF-alpha inhibitors post-infection. Moderate-to-highly immunosuppressive agents with long half-lives such as cyclophosphamide, anti-CD20 and anti-BAFF antibodies, CTLA-4 IgG would be omitted or discontinued [1].

The data published after closure of the survey do not show substantially increased risk of infection or severe course of COVID-19 in AID patients-receiving immunosuppressive and/or biologic treatment [64–66]. A possibly less stringent approach regarding the long-term immunomodulatory therapy than suggested from our respondents might be required.

## Limitations

The results of this survey depict personal therapeutic decision-making perspectives amid COVID-19 pandemics. The heterogeneity of the clinical scenarios and possible (combination) therapies were not explicitly addressed. Some of the respondents had no or limited experience in treating COVID-19 patients so that the statements are partially based on hypothetical considerations. The results are based on participation of relatively large group of specialists; however, a non-response bias has to be taken into account as majority of the addressed experts did not respond. Similarly, the unequal distribution of geographical origin of the respondents may affect the overall result. Thus, our conclusions are preliminary and not meant as treatment recommendations.

## Conclusions

There is a growing evidence that pediatric patients with AID receiving immunomodulatory therapy are not substantially endangered by SARS-CoV-2 beyond the common risk of viral infections. Responses from this survey are largely in line with the evolving findings and endorse continuation of most of the immunomodulatory therapies in case of mild COVID-19. While a severe or complicated course of acute COVID-19 in pediatric population remains rare, the hyperinflammatory state (PIMS-TS/MISC) constitutes a significant clinical challenge and requires continuous attention and further research.

## Supplementary Information

Below is the link to the electronic supplementary material.

Supplementary file1 (DOCX 194 kb)

## Abbreviations

ACR: American College of Rheumatology
ADE: Antibody dependent enhancement
AID: Autoimmune/inflammatory disease
COVID-19: Corona virus disease 2019
FDA: Food and Drug Administration
HCQ: Hydroxychloroquine
IVIG: Intravenous immunoglobulins
JAK: Janus kinase
MERS: Middle east respiratory syndrome coronavirus
SARS: Severe acute respiratory syndrome coronavirus
