# Supplementary material for: Therapeutic approaches to pediatric COVID-19: an online survey of pediatric rheumatologists
Source: Rheumatol Int. 2021 Mar 8;41(5):911–20. doi: 10.1007/s00296-021-04824-4 (PMC7938886; doi:10.1007/s00296-021-04824-4)

**Supplementary Figure 1.** Online survey form.

**Therapeutic approaches to pediatric COVID-19 disease (opinion poll)**

**1. What type of medical setting do you work in?**

university hospital

other hospital

an outpatient setting

**2. In which country do you work?** ___________________________________________________

**3. How long have you been working in the field of pediatric rheumatology?**

< 5 years

5-10 years

> 10 years

**4. Have you treated patients with COVID-19?**

yes

no

**a. If yes, how many patients have you treated so far?**

1 - 5

 > 5

**b. If yes, were these patients**

adults?

children?

**5. For a pediatric COVID-19 inpatient, would you consider off-label medications in addition to standard pneumonia therapy?**

yes

no

**6. Which medications would you consider in pediatric inpatient with COVID-19 (in addition to standard pneumonia therapy)?**  (Multiple entries are possible)
Legend:
(*) Possible criteria for stage II COVID-19 disease: increased respiratory rate, supplemental oxygen requirement, increased pCO2, chest X-ray/CT with bilateral infiltrates, subpleural ground glass pattern or consolidation, lab parameters: ↓ lymphocytes, ↓ thrombocytes, ↑ IL-6, ↑↑ CRP, ↑ PCT, ↑ IL-2R, ↑ ferritin, ↑ LDH, ↑ transaminases, ↑ D-dimers, ↑ Troponin.

|  | **Stage I:** supplemental O2-requirement, but without signs of cytokine storm | **(*) Stage II:** Clinical deterioration: early signs of cytokine storm/ARDS | **Stage III:**Critically ill patients: ARDS/cytokine storm/organ failure |  | I would **NOT** recommend this drug | I do not know |
| --- | --- | --- | --- | --- | --- | --- |
| Anakinra |  |  |  |  |  |  |
| Azithromycine |  |  |  |  |  |  |
| Eculizumab |  |  |  |  |  |  |
| Hydroxychloroquine |  |  |  |  |  |  |
| IVIG |  |  |  |  |  |  |
| JAK inhibitors |  |  |  |  |  |  |
| Lopinavir-Ritonavir |  |  |  |  |  |  |
| Prednisolone ≤ 2 mg/kg/d |  |  |  |  |  |  |
| High-dose prednisolon-    therapy 10-30 mg/kg/d |  |  |  |  |  |  |
| Convalescent plasma |  |  |  |  |  |  |
| Remdesivir |  |  |  |  |  |  |
| Tocilizumab |  |  |  |  |  |  |

Other medications:__________________________________________________________________________

Comments:_______________________________________________________________________ 

**7. How would you approach patients at high risk for severe viral infection, i.e. patients with relevant primary or secondary immunodeficiency or severe cardiopulmonary, renal or neurodegenerative diseases? Would you consider earlier use of the following medications?**(Multiple entries are possible)

Earlier use of antiviral drugs

Earlier use of steroids

Earlier use of oral immunomodulatory drugs

Earlier use of cytokine blockade

Earlier use of immunoglobulins

Earlier use of convalescent plasma

None of the above

Comments:_______________________________________________________________________

**8. Have you treated patients with Pediatric Inflammatory Multisystem Syndrome (PIMS) possibly associated with SARS-CoV-2 infection?**

yes

no

**If yes, could we get in touch with you to learn more about your experience?**

yes

no

**Your email address (optional)** ________________________________________________________

**9. Would you like to share with us your current approach (during the COVID-19 pandemic) to the disease-modifying drugs (DMARDs) in your patients?**

yes

no (jump to the end of the survey)

**10. Do you currently advise your patients to preemptively reduce or discontinue disease-modifying drugs (DMARDS) without these patients displaying any signs of COVID-19 infection (without SARS-CoV-2 positivity nor suspected contact)?**

yes

no

If yes, which medications? _____________________________________________________

**Which immunomodulatory medications would you modify or discontinue for an outpatient patient who tests positive for SARS-CoV-2 and presents with clinical symptoms of a respiratory infection?**

|  | **no change** | | **reduce** | **stop** |
| --- | --- | --- | --- | --- |
| Azathioprine |  |  | |  |
| BAFF blockade (e.g. Belimumab) |  |  | |  |
| B-cell therapy (e.g. Rituximab) |  |  | |  |
| CTLA-4 blockade (e.g. Abatacept) |  |  | |  |
| Ciclosporin / Tacrolimus |  |  | |  |
| Cyclophosphamide i.v. (0,5-1g/m2 monthly) |  |  | |  |
| Dapsone |  |  | |  |
| Hydroxychloroquine |  |  | |  |
| IVIG or SCIG |  |  | |  |
| IL-1 blockade (Anakinra) |  |  | |  |
| IL-1 blockade (e.g. Canakinumab) |  |  | |  |
| IL-6 blockade (e.g. Tocilizumab) |  |  | |  |
| IL-12/-23 blockade (e.g. Ustekinumab) |  |  | |  |
| JAK blockade (e.g. Tofacitinib) |  |  | |  |
| Leflunomide |  |  | |  |
| mTor Inhibitors (e.g. Sirolimus, Everolimus) |  |  | |  |
| Mycophenolate |  |  | |  |
| Prednisolone p.o. ≤ 2 mg/kg/d |  |  | |  |
| Prednisolone p.o. > 2 mg/kg/d |  |  | |  |
| High-dose methylprednisolone i.v. |  |  | |  |
| Methotrexate (≤15mg/m2/Week) |  |  | |  |
| TNF-α blockade s.c.: e.g. Etanercept,Adalimumab |  |  | |  |
| TNF-α blockade i.v.: e.g. Infliximab |  |  | |  |

Other medications _______________________________________________________________

Comments_____________________________________________________________________

General comments _______________________________________________________________

| **Country** | **n (%)** |
| --- | --- |
| USA | 47 (50.5) |
| Germany | 7 (7.5) |
| Brazil | 5 (5.4) |
| Canada | 5 (5.4) |
| Italy | 3 (3.2) |
| United Kingdom | 3 (3.2) |
| Czech Republic | 2 (2.2) |
| Sweden | 2 (2.2) |
| Turkey | 2 (2.2) |
| Australia, Chile, Colombia, France, Greece, Israel, Kenya, Netherlands, Oman, Paraguay, Serbia, Slovenia, Spain, Switzerland | One respondent in each case |
| No data available | 3 (3.2) |

**Supplementary Table 1.** Country of origin of the participating respondents (n = 93).

**Supplementary Figure 2.** Country of origin of the participating respondents, geographical display (n = 93).


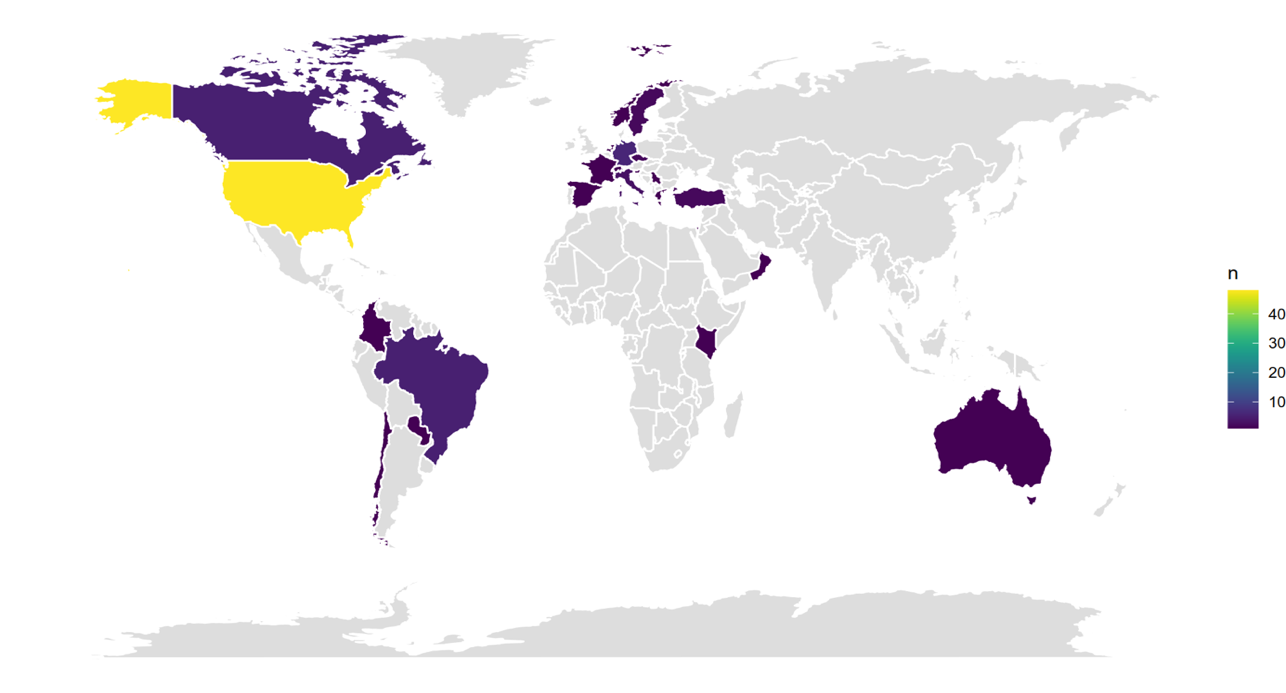

Supplement: Supplementary file 1 — Supplementary file1 (DOCX 194 kb) [file 296_2021_4824_MOESM1_ESM.docx]
